# *In Situ* Single-crystal X-ray Diffraction Studies of Physisorption and Chemisorption of SO_2_ within a Metal–Organic Framework and Its Competitive Adsorption with Water

**DOI:** 10.1021/jacs.3c11847

**Published:** 2024-01-26

**Authors:** Russell M. Main, Simon M. Vornholt, Romy Ettlinger, Philip Netzsch, Maximillian G. Stanzione, Cameron M. Rice, Caroline Elliott, Samantha E. Russell, Mark R. Warren, Sharon E. Ashbrook, Russell E. Morris

**Affiliations:** † EaStCHEM School of Chemistry, Purdie Building, North Haugh, St AndrewsKY16 9ST, U.K.; ‡ Department of Chemistry, SUNY Stony Brook, 100 Nicolls Road, 104 Chemistry, Stony Brook, New York11790-3400, United States; § Diamond Light Source Ltd, Diamond House, Harwell Science & Innovation Campus, Didcot OX11 0DE, U.K.

## Abstract

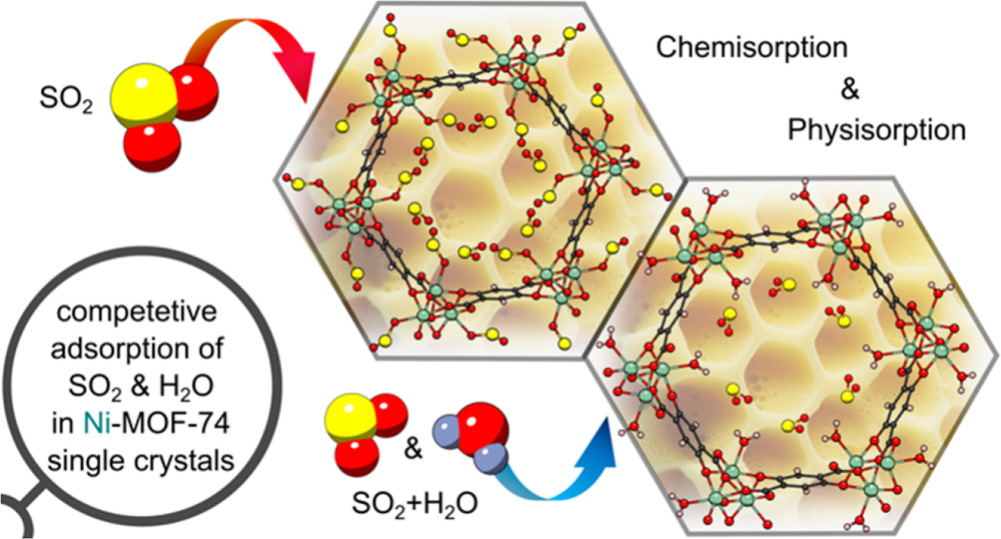

Living on an increasingly polluted planet, the removal of toxic pollutants such as sulfur dioxide (SO_2_) from the troposphere and power station flue gas is becoming more and more important. The CPO-27/MOF-74 family of metal–organic frameworks (MOFs) with their high densities of open metal sites is well suited for the selective adsorption of gases that, like SO_2_, bind well to metals and have been extensively researched both practically and through computer simulations. However, until now, focus has centered upon the binding of SO_2_ to the open metal sites in this MOF (called chemisorption, where the adsorbent–adsorbate interaction is through a chemical bond). The possibility of physisorption (where the adsorbent–adsorbate interaction is only through weak intermolecular forces) has not been identified experimentally. This work presents an *in situ* single-crystal X-ray diffraction (scXRD) study that identifies discrete adsorption sites within Ni-MOF-74/Ni-CPO-27, where SO_2_ is both chemisorbed and physisorbed while also probing competitive adsorption of SO_2_ of these sites when water is present. Further features of this site have been confirmed by variable SO_2_ pressure scXRD studies, DFT calculations, and IR studies.

## Introduction

The capture of pollutants such as nitrogen oxides (NO_*x*_) or sulfur dioxide (SO_2_) from the atmosphere or industrial flue gases is urgently needed.^1−4^ SO_2_ in the troposphere not only leads to the formation of acid rain,^5^ but there is growing evidence of its contribution to chronic illness.^6^ It is produced in large quantities during fossil fuel combustion from sulfur contaminants in the product stream^7^ and is a significant pollutant in flue gas outflow from power stations that can limit the application of potential carbon dioxide (CO_2_) capture and storage devices.^8^ Therefore, materials that can capture or degrade SO_2_ are a major target for research.

The class of metal–organic frameworks (MOFs) holds promise for addressing these challenges. Their structures consist of metal nodes, bound together by organic linkers to create large open lattice frameworks.^9^ MOFs are well-known for their high porosities and exceptional surface areas of up to 10,000 m^2^g^–1^.^10^ Owing to these properties, the use of MOFs as gas storage devices for energetic gases, e.g., H_2_^11^ and CH_4_,^12,13^ as well as carbon capture and storage^14,15^ has already been widely researched.^16^

The MOF-74/CPO-27 family of MOFs is of great interest due to its stable porosity and the high density of open metal sites within its internal pore environment.^17−19^ The M^2+^ metal ions (M = Mg, Ni, Co, Cu, or Zn) are connected by 2,5-dihydroxylterephthalaate linkers (2,5-dhtp) into a porous structure. It crystallizes in the *R*3̅ space group and contains one-dimensional hexagonal channels running along the crystallographic *c*-axis. After thermal activation, these channels contain open metal sites, making MOF-74 a very attractive option for the storage of gases, particularly those that are good ligands that can form a bond to the metal on adsorption (a process termed chemisorption). Henkelis et al. have shown that Mg-MOF-74 is capable of preferential adsorption of SO_2_ over water in a wet gas stream designed to be similar to that found in flue gas.^20^ There have been many computational studies that have shown the high binding energies of SO_2_ to the open metal site of Mg-MOF-74 and that SO_2_ is predicted to bind in preference to water,^4,21−25^ supporting the results observed by Henkelis et al.^20^ However, open metal sites are not the only means by which SO_2_ can be captured by a MOF. For example, Walton et al.^26^ have shown that the binding of different modulators within UiO-66 has a marked effect on its SO_2_ uptake; Parker et al.^27^ showed that functionalized amine groups within Mg_2_(dobpdc) could chemically bind SO_2_; and Smith et al.^28^ have shown that there are a variety of binding sites within MFM-170(Cu). While in the vast majority of spectroscopic and computational studies the possibility of physisorbed sites (i.e., those where the adsorbate-adsorbent interaction does not involve chemical bond formation) has been ignored, very little research has focused on potential secondary binding sites that play a role in competitive coadsorption of gases at the metal site.^29,30^ At the very least, both physisorbed and chemisorbed species are possible; however, no diffraction study has identified the physisorption site for SO_2_ in MOF-74 materials.

To fully understand how gases are adsorbed by MOFs, it is important to know exactly how the gases bind within the frameworks. *In situ* studies of gas adsorption using single crystal X-ray diffraction (scXRD) provides an invaluable tool to improve our understanding of adsorption but is experimentally challenging.^31^ By utilizing the unique brightness and flux of high energy X-rays available at a synchrotron facitly,^32^ it is possible to obtain and model data of high enough resolution to observe gas molecules within the MOF pore environment. In our previous work on NO and CO binding in Ni-MOF-74,^33,34^ we could only observe the chemisorbed binding site as any physisorbed molecules were too disordered over multiple sites. In general, in MOFs the accessible pore volume available for physisorption outweighs that available for chemisorption, even in MOFs like Ni-MOF-74 which has a relatively large number of open metal sites. However, observing a physisorbed site with scXRD is difficult due to the high amount of disorder and there are only a small number of examples, all published in the past few years.^1,28,35^

Here, we present scXRD data obtained at Diamond Light Source (UK) showing SO_2_ binding within an activated Ni-MOF-74 framework and how this is affected by changes in temperature and pressure. Not only is the chemisorbed site located, but at room temperature, a previously unreported physisorbed site has also been observed. We have performed DFT calculations to confirm the energetic properties of SO_2_ binding within this site, as well as the SO_2_ loading of Mg-and Ni-MOF-74 in a humid gas stream to confirm the preferential uptake of SO_2_ over water in both MOFs, as reported by Nenoff et al.^20^

## Results

### *In Situ* scXRD Study Loading Ni-MOF-74 with SO_2_

To study the loading of Ni-MOF-74 with SO_2_, single crystals were synthesized according to the procedure reported elsewhere (Figure S1).^33^ After the crystal was selected, it was first activated at elevated temperatures (450 K) and vacuum (3.3 × 10^–6^ mbar). As observed in previous studies, complete dehydration, i.e., a residual occupancy of M–O_water_ (O_w_) of 0% could not be achieved even after treatment under high vacuum and temperature.^33,34^ Therefore, samples were considered activated when the residual occupancy of O_w_ fell below 10%. It was found that if the activated samples were cooled, the amount of bound water increased even under a (dynamic) vacuum. This suggests that, because the whole gas delivery system cannot be heated, some water remains bound on the cool surfaces of the gas system even when the crystal itself is heated and this water is readsorbed once the crystal is cooled because of the high affinity of water to the Ni site.^30^ To mitigate this to some degree, the crystal was activated at 450 K and then exposed to SO_2_ while it was still at this temperature. It was then cooled to 300 K to observe how the gas binding changes with temperature.

A list of experimental details with corresponding refinement quality factors (*R*_1_) can be found in Table S1, and the full structure determination details have been deposited with the Cambridge Crystal Structure Database as described below.

**Figure 1:**
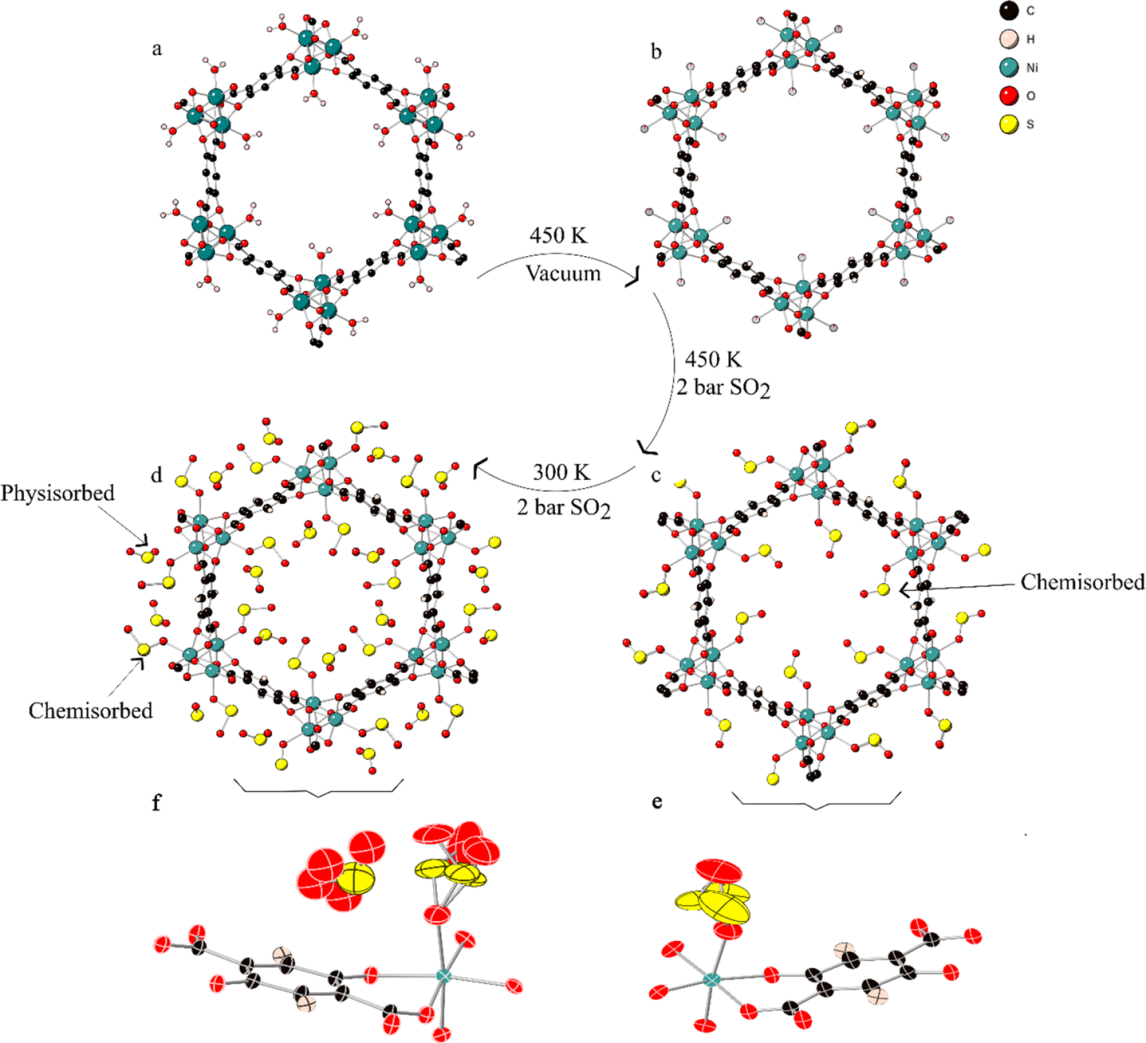
Showing honeycomb channels of Ni-MOF-74, modeled with balls and sticks, under (a) atmospheric pressure and room temperature, (b) 450 K and 3.3 × 10^–6^ mbar with 7.4(11)% O_w_ occupancy, (c) 450 K and 2 bar of SO_2_, with 79(2)% SO_2_ loading, and (d) 300 K, 2 bar SO_2_ pressure with two SO_2_ sites of occupancies 100(2)% chemisorbed and 77.7(19)% physisorbed; and showing the disorder modeling within SO_2_ loaded Ni-MOF-74 with 50% probability ellipsoids, under (e) 450 K and 2 bar SO_2_ and (f) 300 K and the same gas pressure.

The structure of activated Ni-MOF-74 shows the expected *R*3̅ symmetry and honeycomb structure (Figure 1).^36^ Once the observed residual occupancy of O_w_ at 450 K was down to 7.4(11)% (Figure 1b, Supplementary CIF 1), SO_2_ was introduced to the sample at 450 K and 2 bar. Under these conditions, SO_2_ binds to the open metal site, through one of the oxygen atoms, with an occupancy of 79(2)% (Figure 1c, Supplementary CIF 2). The SO_2_ was highly disordered but this could be modeled by splitting the sulfur atom over three sites with restraints on the bond distances to ensure a stable refinement (see Figure 1e and Supporting Information, Supplementary Methods). The second oxygen could only be modeled at one site; however, the refined oxygen occupancy is lower than expected at 16(3)%, indicating that the oxygen atom is in fact distributed over multiple positions around the sulfur atoms and this is merely the most likely position in which it can be found. The S–O bonds were subject to a bond distance (SHELXL DFIX) restraint and range between 1.416(19) and 1.448(14) Å, similar to the free SO_2_ bond length.^37^ The Ni–O bond was allowed to be refined unrestrained to 2.229(8) Å.

Reducing the temperature to 300 K (Figure 1d, Supplementary CIF 3) while maintaining the same SO_2_ pressure had two effects. First, it increased the amount of metal bound SO_2_ to 100(2) %. The lower temperature decreased the disorder in the SO_2_ as seen by the reduction in the anisotropic parameters and the ability to better model the disorder in the outer oxygen atoms, Figure 1f. The Ni–O bond length reduced to 2.161(5) Å, indicating a stronger bond with the guest. The S–O unrestrained bond lengths of the metal bound SO_2_ molecules were in the range 1.41(2)–1.46(2) Å, a broad range but close to that of free SO_2_.^37^ The secondary S–O bonds were restrained and ranged between 1.392(15) and 1.44(2) Å (Figure 1f).

Second, the reduction in temperature led to some physisorbed SO_2_. This SO_2_ is, as expected, highly disordered but can be modeled with one sulfur atom (occupancy of 77.7(19) %) and four oxygen atoms. The S–O bond lengths were restrained with a DFIX restraint and vary between 1.379(17) and 1.426(17) Å (Figure 1f).

It was not possible to model any further physisorbed SO_2_ sites, but by calculating the free pore volume and the excess electron density found within using the Olex mask command with a 1.2 Å probe, an estimate of how much unmodelled SO_2_ in the pore can be obtained. The calculated masks show the expected trend for the pore volume. The activated sample had a free pore volume of 2121 Å^3^ per unit cell, and on adding chemisorbed SO_2_, this reduced to 1014 Å^3^ per unit cell. Cooling the sample (with chemisorbed and physisorbed SO_2_ modeled) caused a further reduction to 393 Å^3^ per unit cell. For comparison, the free pore volume of a theoretical fully dehydrated sample is 2365 Å^3^ per unit cell; therefore, at 300 K, the modeled SO_2_ takes up 83% of the available pore volume. The calculated electron densities, which are useful proxy for any free gas molecules within the pore, showed a different trend. The activated sample had 0.014 e^–^ per Å^3^, the SO_2_ loaded sample had 0 e^–^ per Å^3^ (at 450 K) and cooling it increased it back to 0.107 e^–^ per Å^3^. These data indicate that at 450 K there is no physisorbed SO_2_ at all, while at 300 K, there is the modeled physisorbed site plus approximately one unmodeled SO_2_ molecule per unit cell loosely bound within the free pore volume at 300 K.

The free volume available in the activated sample can theoretically hold 29 SO_2_ molecules per unit cell (using a kinetic diameter of 3.6 Å).^38^ Our model suggests the maximum loading would be 12 chemisorbed SO_2_ molecules, 12 physisorbed SO_2_ molecules, and 5 free SO_2_ molecules making 29 per unit cell overall. At 2 bar and 300 K, we can model 22 of these molecules.

### DFT Calculations

The physisorbed binding site for SO_2_ modeled above has, as far as we are aware, never been observed or predicted computationally in any previous study, though similar sites have been modeled for other gases.^39,40^ Therefore, it was desirable to compute whether this site was energetically favorable in other MOF-74 systems. To do this, we performed a DFT calculation of the binding energies of the two sites within Mg-MOF-74. The magnesium sample was chosen to match the previous work done by Nenoff and co-workers.^20^ The purely metal bound SO_2_ had a binding energy of 89.5 kJ mol^–1^; a similar value to that found in the literature for this system (Table S2).^21−23^ Modeling the chemisorbed and physisorbed sites produced an additional binding energy of 42.6 kJ mol^–1^. In models that contain nonmetal bound SO_2_, similar binding energies have been observed (Table S2).^35^

**Figure 2:**
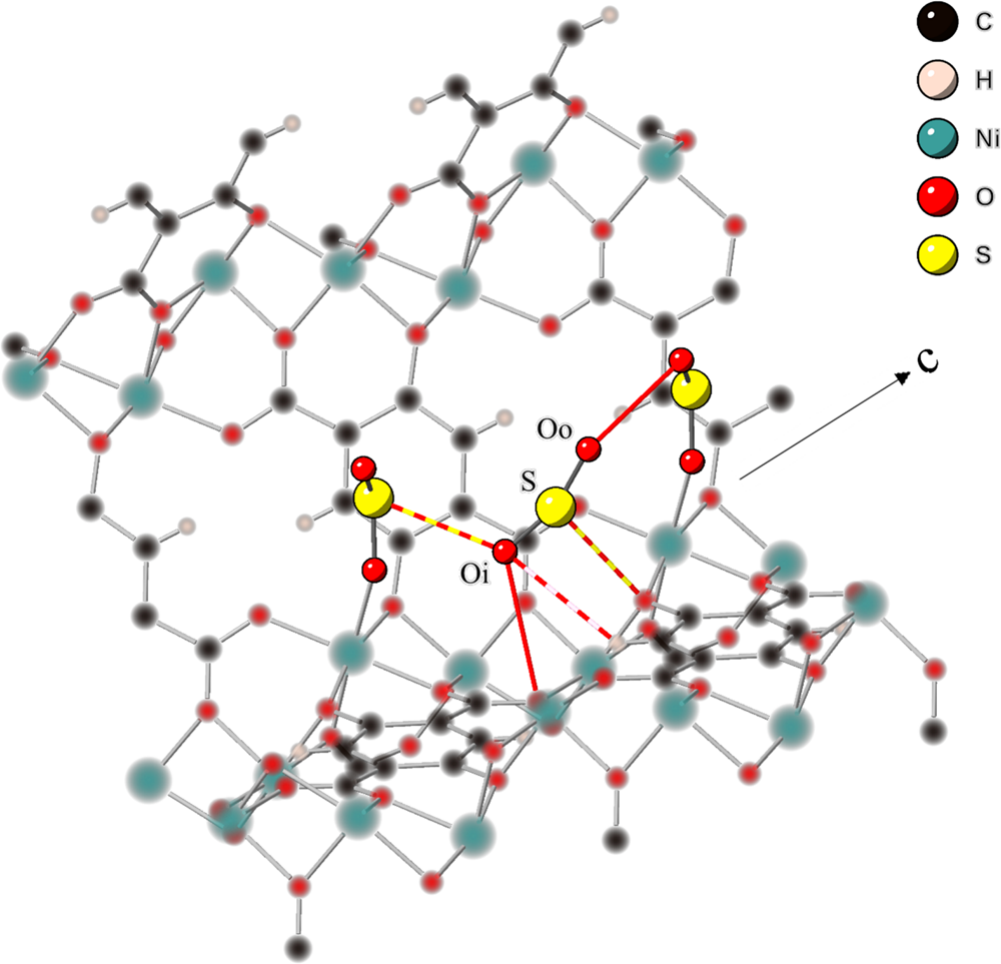
Schematic, with 40% space filling spheres, showing the interactions marked in Table 1 between the physisorbed SO_2_ and the rest of the framework. The color of each line indicates which atoms are involved in the interaction. The framework is shown blurred for ease of view with an arrow marking the direction of the *c* axis and pore direction.

By comparing the DFT optimized structure with that modeled from the scXRD data, it is possible to observe which interactions are likely to be most important in binding the second SO_2_. Table 1 shows the relevant interactions and compares the differences between the atomic distances in structures from DFT and scXRD. Some of the interactions are highlighted and shown in Figure 2. It is possible to distinguish between the two physisorbed oxygen positions as one points in toward the wall of the framework (labeled O_i_) the other points out into the internal pore (labeled O_o_).

**Table 1:** Table Showing Selected Atomic Distances from Structures Optimized Using DFT Calculations and Their Equivalent Distances within the scXRD Data Set (The Distances Marked * Are Shown in Figure 2)

bond type	distance in DFT/Å	equivalent distance in scXRD/Å
O_i_ -- metal bound S *	2.907	2.975(35)
O_i_ -- framework H *	2.964	3.068(28)
O_o_ -- other physisorbed S	3.307	3.308(46)
S -- framework O *	3.112	3.0688(87)
O_i_ -- outer O in metal bound SO_2_	3.250	3.012(68)
O_o_ -- outer O in metal bound SO_2_ *	3.278	2.763(73)
S -- O in metal bound in SO_2_	3.293	3.484(10)
O_i_ -- framework O *	3.462	3.261(38)
S -- framework aromatic Cs	3.564	3.506(10)
O_o_ -- framework H	3.757	2.996(49)

Figure 2 shows that two main interactions taking place. The first is between the SO_2_ and the framework, with the aromatic hydrogen and a carboxylic oxygen of the 2,5-dhtp linker both within 3 Å of the SO_2_. Second, there is the interaction between the physisorbed SO_2_ and the metal bound SO_2_ with both O_i_ and O_o_ binding to a different metal bound SO_2_ along the length of the pore with interaction distances around 3 Å. This shows that this binding pocket is only accessible after SO_2_ has bound to the metal. It may also further explain why this site was unoccupied at 450 K. Not only does the high temperature start to overcome the binding energy, but the increased motion of the metal bound SO_2_ will disrupt the favorable binding interaction. The presence of only long-range binding interactions along with the low enthalpy of adsorption confirm that this is a physisorbed site.^41^

### Variable Pressures of SO_2_ in Ni-MOF-74

**Figure 3:**
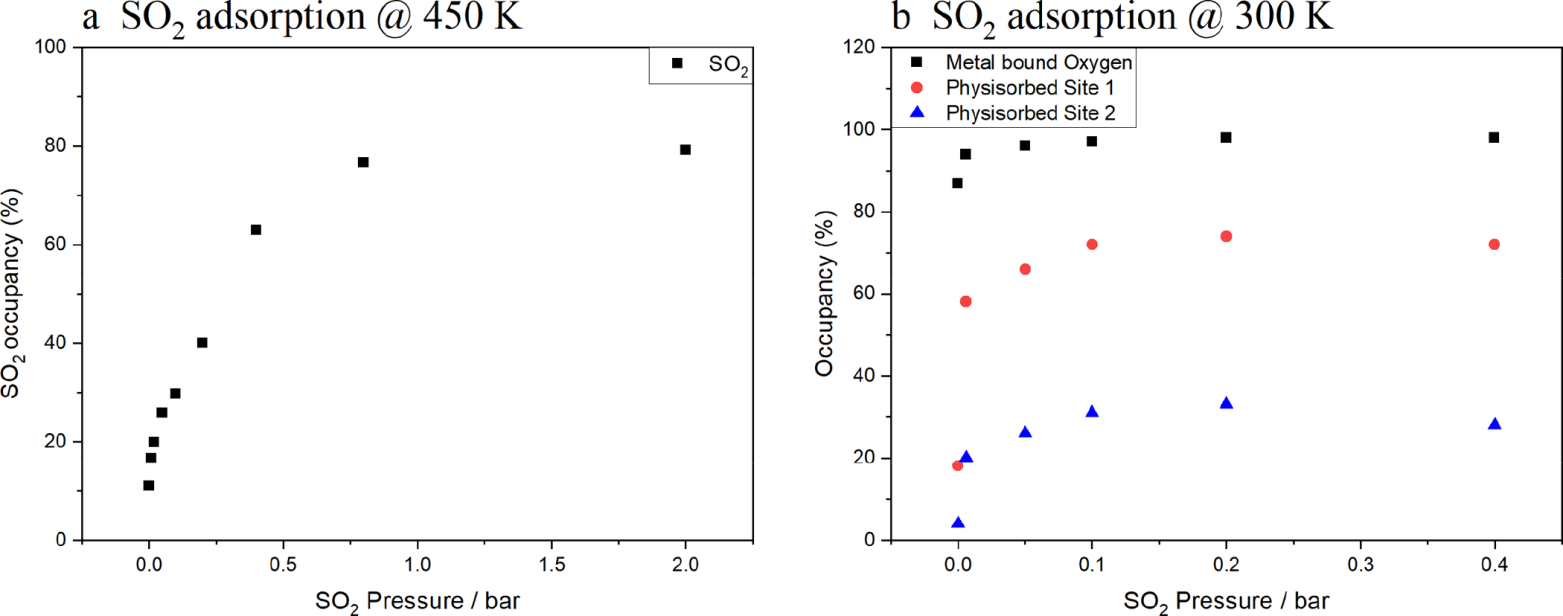
Plot showing how the occupancy of (a) metal bound SO_2_ in Ni-MOF-74 increases as a function of pressure at 450 K, and (b) the three different SO_2_ binding regions (metal bound: black, physisorbed site 1: red, physisorbed site 2: blue) changes with pressure at 300 K.

To further investigate SO_2_ binding within Ni-MOF-74, the MOF was loaded with SO_2_ at different gas pressures and the structure was analyzed using scXRD. At 450 K, SO_2_ only binds to the open metal site and the occupancy increases with pressure similar to type I isotherm behavior (Figure 3a, Supplementary CIF 4). The SO_2_ loading plateaus above 0.8 bar. At this temperature, it is not possible to model any physisorbed SO_2_ molecules and there is no additional electron density within the pore environment, indicating that SO_2_ only binds to the metal site.

At 300 K, a different behavior was observed (Figure 3b, Supporting Information, CIF 5). Initially, the readsorption of water on cooling from the activation temperature of 450 to 300 K while under vacuum was significant, with the O_w_ occupancy increasing from 8 to 85%. As described earlier, this water uptake is likely due to condensation on the gas rig being readsorbed by the MOF. However, this gives us an excellent system with which to probe the effect of excess SO_2_ adsorption in the presence of some water. Once SO_2_ was introduced, it initially binds to the remaining free metal sites with the level of O_w_ reaching 94% at only 0.006 bar of SO_2_. It then occupies the physisorbed site described earlier and then the center of the pore. Significantly, SO_2_ appears to readily bind into the physisorbed site even when there is a large proportion of water bound to the metal instead of SO_2_. The three binding modes all follow a type I isotherm on increasing SO_2_ pressure (Figure 3b) with the level of O_w_ plateauing at 0.05 bar and the two physisorbed sites at 0.1 bar. At pressures of ≤0.4 bar, it is best to model these sites with three constrained oxygen atoms (see Supporting Information and Figure S3) to improve comparability between the data sets. The constraints necessary for the successful modeling of the SO_2_ molecules at low pressure means that we cannot be confident that small changes (for example, the slight reduction in occupancy of the physisorbed sites at 0.4 bar) are real effects as the estimated uncertainties on the occupancy (which can all be found in the CIF files) are necessarily quite large for these points.

**Figure 4:**
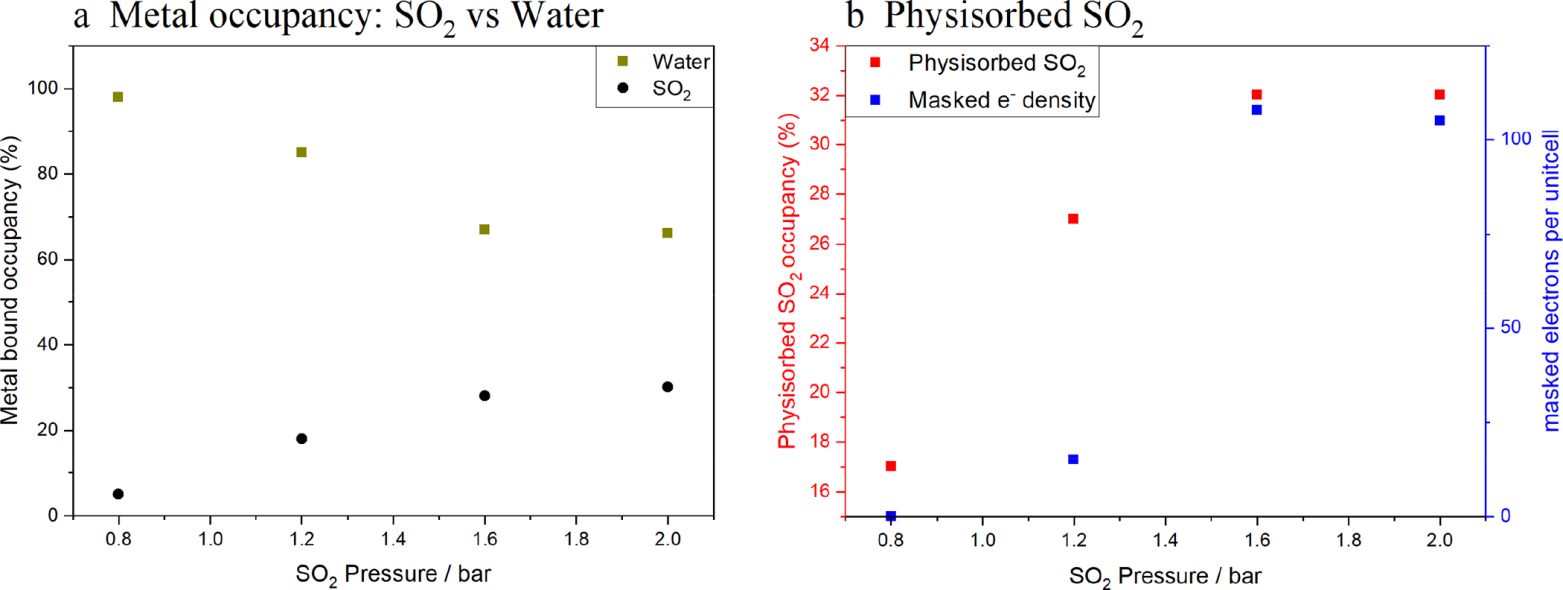
Plots showing how SO_2_ occupancies change with increasing pressure: (a) showing how metal bound occupancies change (water: olive, SO_2_: black), (b) showing how physisorbed SO_2_ occupancy increases (physisorbed SO_2_: red, masked electron density [measurement of unmodelled SO_2_]: blue).

At pressures higher than 0.4 bar, the SO_2_ molecules are modeled without constraints and the remaining pore environment electron density calculated. It is possible to observe SO_2_ replacing the metal bound water as the SO_2_ pressure is increased above 0.8 bar (Figure 4a); below 0.8 bar, no replacement of metal-bound water by SO_2_ is seen. We can also observe that the physisorbed occupancy increases above 0.8 bar of SO_2_ (Figure 4b) showing that this site is filled next. The electron density calculated using the mask shows that unmodeled SO_2_ increases after about 1.2 bar. Applying a dynamic vacuum after loading the sample with SO_2_ at 300 K was unable to remove the SO_2_, with both the metal bound and physisorbed occupancies remaining similar after 50 min (Figure S4).

These data show the subtle features of water/SO_2_ competitive adsorption. Under high enough pressure, some metal bound water is replaced, but at 2 bar of SO_2_ pressure, the extent of replacement is still only ∼30%. We can therefore see the importance of activating Ni-MOF-74 before it is used for SO_2_ capture, especially at low pressures. However, there is some availability for SO_2_ physisorption even at lower pressures, and so this may be an important mechanism in the removal of SO_2_ from flue gas.

### SO_2_ Loading in a Humid Atmosphere

In order to validate the comparison of Mg- and Ni-MOF-74 the SO_2_ loading in humid conditions was performed for both MOFs. To do this, we built a modified gas loading rig^20^ whereby gaseous SO_2_ was produced from the reaction of Na_2_SO_3_ and H_2_SO_4_ under aqueous conditions and passed over Mg- and Ni-MOF-74 with a N_2_ as carrier gas (Figure S5). The MOFs were not fully activated in order to make the experiment more applicable to an industrial setting, where the high energy and cost of fully activating and storing MOF-74 would be prohibitive. Instead, the MOF samples were dried at 140 °C under a N_2_ flow in order to remove the majority of the solvent as predicted by thermal gravimetric analysis (Figure S2). This likely results in a MOF with a high amount of metal bound water but with available physisorbed binding environments, which we know from the scXRD studies are important in low temperature SO_2_ binding. Infrared spectroscopy (IR) was used to observe whether any SO_2_ is present (Figure S6a,b) and powder X-ray diffraction (PXRD) to observe how this affected the crystal structures (Figure S7).

**Figure 5:**
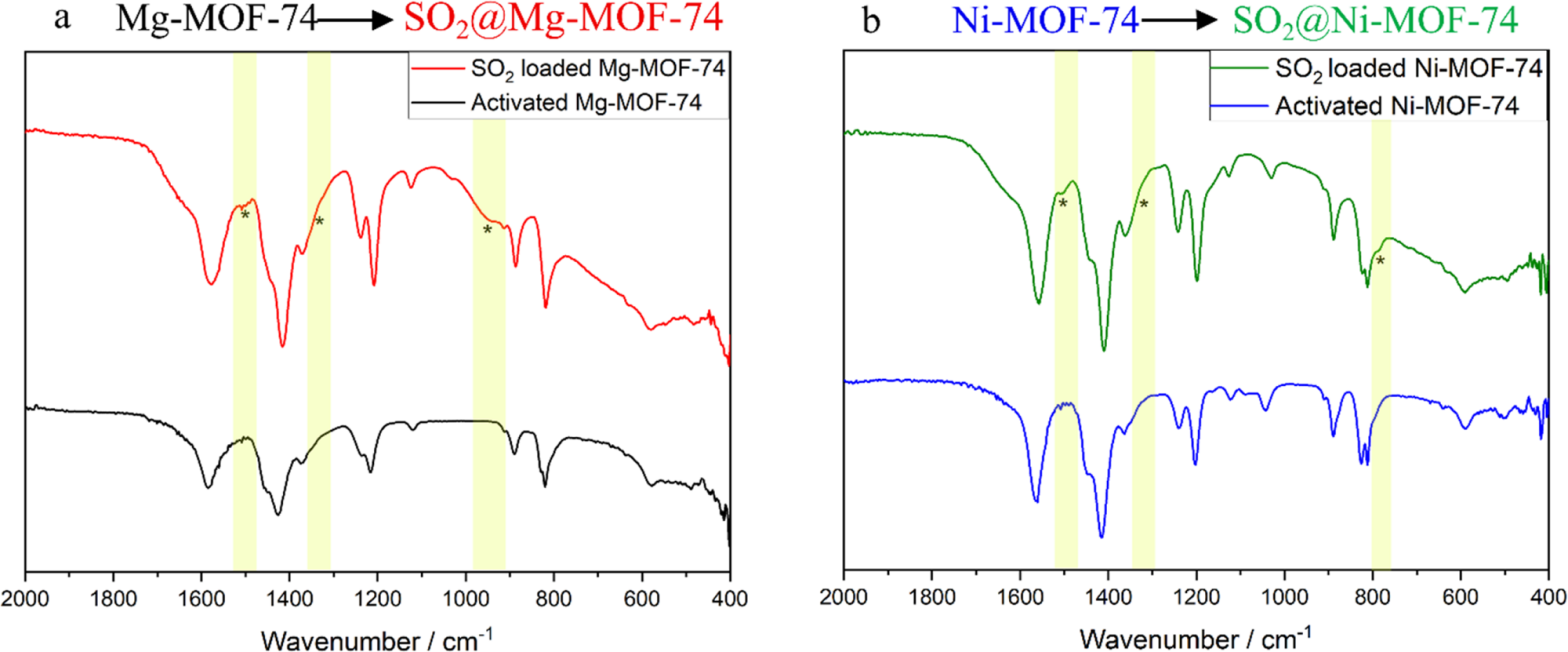
Comparison of IR-spectra of Mg-MOF-74 (a) and Ni-MOF-74 (b) before and after SO_2_ loading. The marked regions in the IR spectra highlight the changes associated with SO_2_.

Exposing an activated sample of Mg-MOF-74 to the wet SO_2_ gas stream caused the sample to adsorb SO_2_ and water. This can be seen in the IR spectra in Figure 5a and Figure S6, in which the appearance of a broad peak around 3300 cm^–1^ shows that water has bound and the marked peak around 950 cm^–1^ shows that SO_2_ is present. There are further smaller peaks at 1500 and 790 cm^–1^ that also arise from SO_2_ and have been marked along with a shoulder at 1330 cm^–1^ that Henkelis et al. also associated with SO_2_ loading.^20^

However, adsorbing SO_2_ caused a small amount of structural change within the MOF. If Mg-MOF-74 was exposed to larger amounts of SO_2_, it decomposed into MgSO_3_ (H_2_O)_6_ and a small amount of an unknown phase likely due to the production of H_2_SO_3_ when the SO_2_ and water react (Figure S7). However, at the low SO_2_ concentrations used here, there was no sign of any impurities forming. Henkelis et al.^20^ also observed that Mg-MOF-74 would degrade after prolonged exposure within their SO_2_ atmosphere.

Ni-MOF-74 was also capable of adsorbing SO_2_ along with water, as seen in Figure 5b and Figure S6, with the marked bands at 790, 950, and 1500 cm^–1^ all indicating the presence of SO_2_ with the shoulder at 1330 cm^–1^ again difficult to observe in this data set. Experimentally, we found that Ni-MOF-74 was more stable in the SO_2_ flow than its Mg-analogue: when using the same loading conditions, Ni-MOF-74 was stable, while Mg-MOF-74 degraded and was only stable at reduced SO_2_ concentrations (Figure S7 and Methods). This enhanced stability has been seen before in water adsorption^42,43^ and can be explained by the lower lability of the Ni ion.^44^ Therefore, even though computational studies show Mg-MOF-74 has a stronger binding affinity with SO_2_ than Ni-MOF-74,^22,25^ the enhanced stability of Ni-MOF-74 may make it more viable as SO_2_ capture agent within a real world setting such as a power station outflow.

## Discussion

In this work, we used high energy and flux scXRD data to reveal the existence of a previously unknown binding site for SO_2_ within Ni-MOF-74 that plays an important role in SO_2_ adsorption at room temperature. DFT calculations for Mg-MOF-74 confirm the thermodynamic stability of binding SO_2_ in this site in the MOF and provide insight into the important interactions that create the binding pocket. SO_2_ loading experiments confirm that Mg- and Ni-MOF-74 were both capable of adsorbing SO_2_ from an aqueous SO_2_ gas stream verifying the preferable binding of SO_2_ over water. Our results also showed that Ni-MOF-74 has greater stability within the acidic gas stream, indicating its enhanced suitability for the prolonged exposures necessary for a SO_2_ capture device.

This study highlights the importance of combining practical experiments, DFT calculations, and the use of high quality *in situ* scXRD experiments. Only their combined use could prove that the long-standing assumption that only the open metal sites in the MOF-74 family are suitable for adsorbing gases is not entirely accurate. We hope that this will lead to renewed interest, not only in the abilities of the MOF-74 family and its application in pollution capture devices but also the use of *in situ* scXRD to provide real data from which to build more accurate computer models.

## Methods

### Synthesis

Single crystals of Ni-MOF-74 were synthesized using the procedure produced by Vornholt et al.^33^ Nickel acetate tetrahydrate (1 mmol) was dissolved in water (30 mL) and added to a Teflon liner (50 mL). 2,5-Dihydroxyterephthalic acid (0.5 mmol) and 4,6-dihydroxyterephthalic acid (0.5 mmol) were added to the liner, and the mixture was left to stir for 15 min. The liner was then capped, sealed in an autoclave, and placed in the oven for 3 days at 130 °C. Yellow-brown, rectangular rods of Ni-MOF-74 were obtained after filtration.

Bulk Ni-MOF-74 was synthesized using the following procedure. 2,5-dihydroxyterephthalic acid (1.7 mmol) was added to 5.1 mL of 1 M NaOH with stirring at 50 °C. Separately, nickel acetate tetrahydrate (3.4 mmol) was added to 5.1 mL of water with stirring, also heated to 50 °C. The linker solution was added dropwise to the Ni solution once both were fully dissolved. The solution is stirred at 50 °C for 4 h and then filtered to produce a yellow powder of Ni-MOF-74. The powder was partially activated by heating to 140 °C under a N_2_ atmosphere for 5 h.

Mg-MOF-74 was synthesized using the following procedure. 2,5-dihydroxyterephthalic acid (3.5 mmol) was dissolved in 1 M NaOH at 50 °C (13.8 mL). Separately, magnesium acetate tetrahydrate (6.8 mmol) was dissolved in ethanol (22.7 mL) at 50 °C. The magnesium solution was added quickly to 2,5-dhtp solution and all stirred at 50 °C for 1 h before cooling and filtering to produce a yellow powder of Mg-MOF-74. The powder was partially activated by heating to 140 °C under a N_2_ atmosphere for 5 h.

### Single Crystal Experiment

*In situ* gas cell diffraction experiments on single crystals were carried out on a four-circle Newport diffractometer equipped with a Eiger 4 M detector in I19–2 beamline, Diamond Light Source. A wavelength of 0.48590 Å (Ag K-edge) was utilized to give a complete data set from a single 340 degree phi sweep (1700 images, 0.2 deg/image). The selected crystal was mounted with a MiTeGen mount (50 μm) and were secured with a nondiffracting two component epoxy glue (LOCTITE DOUBLE BUBBLE). Care was taken to use as little glue as possible to avoid blocking any channels and ensure good gas transport through the crystal. For gas cell experiments, the crystal mount was inserted into a preassembled gas cell, with super glue used to hold the mount securely in place in the gas cell capillary. The gas cell was then sealed using the Swagelok mechanism and leak tested. The activation temperature was 450 K, with a heating ramp of 360 K/h, in vacuo (3.1 × 10^–6^ mbar at the pump). A data collection at 300 K of the activated systems was obtained for comparison purposes. Activated crystals were exposed to 2.0 bar of the SO_2_ gas at 450 K and data were collected after 30 min exposure. The sample was then cooled to 300 K at the same gas pressure, and another data set was collected. After the crystal had been exposed to the gas for another 30 min, an additional data set was obtained to monitor gas uptake.

To create gas adsorption isotherms, a crystal was first activated at 450 K in vacuo (3.1 × 10^–6^ mbar at the pump). The crystal was then either kept at 450 K or the temperature was reduced to 300 K. The SO_2_ pressure was then increased incrementally and a scan was taken after 5 and 30 min to monitor the gas uptake. The sample loaded at 300 K was then subjected to a dynamic vacuum at 300 K and regular scans were taken to monitor any SO_2_ release.

Data collections were setup using the generic data acquisition (GDA) software and were processed using CrysAlisPro^45^ or xia2^46^ with DIALS^47^ routines. Subsequently, Olex2 GUI^48^ (with shelXT^49^ as solution and shelXL^50^ as refinement tool) was used for structure solution and refinement, respectively. Crystal structures were visualized using the CrystalMaker software kit.^51^ Special refinement details can be found in the supplementary methods.

### DFT Calculations

DFT calculations were performed with the CASTEP code (version 20.11),^52^ using the PBE^53^ functional (with the semiempirical dispersion correction scheme of Tkatchenko and Scheffler),^54^ core–valence interactions described by ultrasoft pseudopotentials^55^ and accounting for relativistic effects using ZORA.^56^ A planewave cut off energy of 60 Ry was used, with the first Brillouin zone sampled by a Monkhorst–Pack^57^ grid with spacing of 0.04 2π Å^–1^. Structural models of activated Mg-MOF-74, and models loaded with 18 and with 36 SO_2_ molecules per unit cell (obtained from scXRD) were geometry optimized, with a geometry optimization energy tolerance of 1 × 10^–4^ eV per atom and an electronic structure energy tolerance of 1 × 10^–9^ eV per atom. All atomic coordinates and unit cell parameters were allowed to vary. To compute the relative energy change associated with SO_2_ loading an additional calculation was also performed for one molecule of SO_2_ optimized in an empty unit cell (with *a* = 25.7523 Å, *b* = 25.7523 Å, *c* = 6.8065 Å, α = 90°, β = 90° and γ = 120°).

### SO_2_ Loading Experiment

SO_2_ was produced by the dropwise addition of a H_2_SO_4_ solution (12% H_2_SO_4_ in water) to an aqueous solution of Na_2_SO_3_ (2 g for Ni-MOF-74, 250 mg for Mg-MOF-74 both in 6.5 mL water). The resultant gas was passed over the dried MOF (70 mg) using a dry N_2_ gas stream which was then bubbled into 500 mL of water in order to remove any excess SO_2_. A schematic of the experimental setup is provided within the Supporting Information and Figure S5.

## Supporting Information Available

Refinement details for each structure presented; PXRD patterns of Ni-MOF-74 single crystals synthesized and predicted; literature values of DFT derived binding energies of SO_2_ in MOFs; TGA data for Ni-MOF-74; full IR spectra of Ni/Mg-MOF-74 with and without SO_2_; PXRD patterns of Mg-MOF-74 after exposures to different concentrations of SO_2_; image of the setup used to load MOFs with SO_2_. The X-ray crystallographic coordinates for structures reported in this study have been deposited at the Cambridge Crystallographic Data Centre (CCDC), under deposition numbers 2288606–2288608, 2288611–2288618, 2288673–2288683. These data can be obtained free of charge from The Cambridge Crystallographic Data Centre via www.ccdc.cam.ac.uk/data_request/cif. The following documents are provided free of charge: Supporting Information, Supplementary CIF 1- Dehydrated Ni-MOF-74 @ 450 K, Supplementary CIF 2- SO_2_ loaded Ni-MOF-74 @ 450 K, Supplementary CIF 3- SO_2_ loaded Ni-MOF-74 @ 300 K, Supplementary CIF 4- SO_2_ loaded Ni-MOF-74 @ 450 K and various pressures, Supplementary CIF 5- SO_2_ loaded Ni-MOF-74 @ 300 K and various low pressures, Supplementary CIF 6- SO_2_ loaded Ni-MOF-74 @ 300 K and various high pressures. The research data supporting this publication can be accessed at https://doi.org/10.17630/ab9867ee-07d1-4c9a-aea4-aa73975f1dbf

## ABBREVIATIONS

MOF: metal–organic framework
SO_2_: sulfur dioxide
CO_2_: carbon dioxide
NO: nitric oxide
DFT: density functional theory
CPO: coordination polymer of Oslo
PXRD: powder X-ray diffraction
scXRD: single crystal X-ray diffraction
dhtp: dihydroxyterephthalic acid
