# Supplementary material for: In Situ Single-crystal X-ray Diffraction Studies of Physisorption and Chemisorption of SO2 within a Metal–Organic Framework and Its Competitive Adsorption with Water
Source: J Am Chem Soc. 2024 Jan 26;146(5):3270–8. doi: 10.1021/jacs.3c11847 (PMC10859936; doi:10.1021/jacs.3c11847)
Supplement: Supplementary file 1 — ja3c11847_si_001.pdf [file ja3c11847_si_001.pdf]

In situ single-crystal X-ray diffraction studies of physisorption and chemisorption of SO_2 within a metal-organic framework and its competitive adsorption with water.

Supplementary Information

Russell M. Main, [1*] *Simon M. Vornholt,*[2] *Romy Ettlinger,* [1] *Philip Netzsch,* [1] *Maximillian G. Stanzione,* [1] *Cameron M. Rice,*[1] *Caroline Elliott,*[1] *Samantha E. Russell,*[1] *Mark R. Warren,*[3] *Sharon E. Ashbrook* [1] *and Russell E. Morris* [1*]

[1] EaStCHEM School of Chemistry, Purdie Building, North Haugh, St Andrews KY16 9ST, UK;

[2] Department of Chemistry, SUNY Stony Brook, 100 Nicolls Road, 104 Chemistry, Stony Brook, NY 11790-3400, USA

[3] Diamond Light Source Ltd, Diamond House, Harwell Science & Innovation Campus,

Didcot, OX11 0DE, UK.

Supplementary Methods

All structural data sets were indexed with CrysAlisPro 1.171.41.93a.,[1] variable pressure samples were indexed with DIALS 3.6.2-g16e93f55b-release.[2] Visualisation was performed using Olex2 1.5 GUI[3] and ShelXT 2014/4[4] for solving and ShelXL 2018/3[5] for refinement.

For the activated (dehydrated) sample, the water bound oxygen was constrained so the Uiso was of the same order as the metal to which it was bound, and was kept isotropic. This was to allow easy comparison of different samples during dehydration without contribution from the pore environment.

For the SO_2 loaded sample at 450 K all the SO bonds were subject to a DFIX restraint of 1.43 Å the gas bond length.[6] All the atoms within the SO_2 were also subject to a SIMU restraint of strength 0.16 over a distance of 2.7 Å. The sulphur atoms were subject to a SUMP restraint so that their total occupancy was within error of the metal bound oxygen atom. The outer oxygen atom was not so restrained as it will be to disordered for a SUMP restraint to be appropriate.

For the SO_2 loaded sample at 300 K. The metal bound SO_2 was subject to a number of restraints. The bond length of the sulphur atoms to the outer oxygen atoms were subject to a DFIX restraint of c. The sulphur atoms were subject to a SIMU restraint with the metal bound oxygen atom of strength 0.08 and distance 2.7 Å as well as a SUMP restraint similar to that used at 450 K. The outer oxygen atoms had a separate SIMU restraint of the same intensity. The physisorbed SO_2 was also restrained with a DFIX restraint of 1.43 Å and a SIMU restraint of 0.04 over 2.7 Å with the oxygen atoms kept isotropic. The oxygen atoms were subject to a SUMP restraint so that their occupancies added up to twice the sulphur occupancy.

In order to calculate free pore volume and electron density the Olex2 mask command was used.[7] Standard parameters (1.2 Å probe) were used. The mask was not used on the published structures, but as a calculation after refinement was finished. The total, theoretical, dehydrated pore volumes were calculated in Olex2 by removing any metal bound oxygen and calculating porosity with standard parameters giving a value of 2364.7 $Å^3$ per unit cell.

In order to calculate the number of gas molecules free in the pore from the observed electron densities the following calculation was performed. The activated e^- density was taken from the gas loaded e^- density to remove any input from disorder within the structure. This corrected value was divided by 32 to obtain the SO_2 molecular density, then multiplied by the free pore volume to obtain the number of SO_2 molecules per unit cell.

For the variable pressure studies different models were used to compare the data. For these data sets comparability between data sets was prioritised over accuracy of the model.

For the loading at 450 K only SO_2 on the metal site could be located. At pressures $\leq$ 0.05 bar only the metal site oxygen could be modelled, this was done without restraint. Above 0.05 bar sulphur could also be modelled, this site was highly disordered but modelled with only one large sulphur atom which was restrained with a SIMU restraint of strength 0.04. In all points the metal bound oxygen occupancy was taken as the SO_2 occupancy. The data point for 2 bar is taken from the structure outlined above.

For the loading at 300 K up to 0.4 bar a 3 oxygen model was used. This consisted of 3 oxygen environments within the pore (Figure S3). The first was metal bound and was unrestrained, anisotropic and with riding hydrogens. The second two sites sat within the pore, both were constrained to be isotropic with a Uiso of 0.05 without riding hydrogens. The oxygen nearest the framework, a similar position to the physisorbed SO_2 site, was named phys 1. The second site further into the pore was labelled phys 2. At higher pressures a more accurate model could be used, though not as accurate as the structural model described above. The metal bound SO_2 was modelled with 2 S sites and 2 outer O sites.

The sulphur atoms were subject to a SIMU restraint with the metal bound oxygen of strength 0.04 and distance 1.7 Å. The oxygen atoms were constrained to be isotropic with a Uiso of 0.1. The metal bound oxygen and the water oxygen were subject to a eadp constraint. In order to estimate the relative SO_2: water ratio SUMP restraints were used so that the total occupancy on the metal site was within 0.05 s.d. of 1 and the total sulphur occupancy was similar to the SO_2 metal bound oxygen amount. The S-O bond lengths were subject to a dfix restraint of 1.43 Å the gas bond length.[6] The physisorbed SO_2 was modelled with 1 sulphur and 2 oxygen atoms all constrained isotropic with a Uiso of 0.05. The occupancy of all 3 atoms was constrained to be equal.

Supplementary Table 1: Containing information on temperature, loading and R_1 values for each dataset presented.

Sample	Temp / K	Loading	R_1	CCDC number
Ni-MOF-74 dehydrated structural	450	7.4(11) % water	9.98	2288608
Ni-MOF-74 SO_2 loaded structural	450	79(2) % SO_2	9.38	2288606
Ni-MOF-74 SO_2 loaded structural	300	100(2) % metal bound SO_2 77.7(19) % physisorbed SO_2	9.39	2288607
Ni-MOF-74-SO_2 variable P 0 bar	450	12(2) % metal O	8.14	2288611
Ni-MOF-74-SO_2 variable P 0.009 bar	450	16.7(14) % metal O	7.21	2288612
Ni-MOF-74-SO_2 variable P 0.02 bar	450	19.8(15) % metal O	7.68	2288613
Ni-MOF-74-SO_2 variable P 0.05 bar	450	25.8(16) % metal O	7.41	2288614
Ni-MOF-74-SO_2 variable P 0.1 bar	450	26.8(17) % metal O	8.40	22288615
Ni-MOF-74-SO_2 variable P 0.2 bar	450	40.5(17) % metal O	8.23	2288616
Ni-MOF-74-SO_2 variable P 0.4 bar	450	63(2) % metal O	10.37	2288617
Ni-MOF-74-SO_2 variable P 0.8 bar	450	79(2) % metal O	14.15	2288618
Ni-MOF-74-SO_2 variable P 0 bar	300	88.9(17) % Metal O, 18.8(14) % phys-1, 4.5(14) % phys-2	7.64	2288678
Ni-MOF-74-SO_2 variable P 0.006 bar	300	94.4(16) % Metal O, 58.9(13) % phys-1, 20.2(13) % phys-2	8.11	2288679
Ni-MOF-74-SO_2 variable P 0.05 bar	300	96.4(17) % Metal O, 67.2(14) % phys-1, 26.1(14) % phys-2	7.88	2288680
Ni-MOF-74-SO_2 variable P 0.1 bar	300	97.3(16) % Metal O, 72.2(14) % phys-1, 31.1(14) % phys-2	7.74	2288681
Ni-MOF-74-SO_2 variable P 0.2 bar	300	98.8(16) % Metal O, 75.0(14) % phys-1, 4.5(14) % phys-2	7.68	2288682
Ni-MOF-74-SO_2 variable P 0.4 bar	300	97.9(17) % Metal O, 72.0(15) % phys-1, 23.2(14) % phys-2	8.05	2288683
Ni-MOF-74-SO_2 variable P 0.8 bar	300	7(5) % Metal bound SO_2, 22.9(6) % physisorbed SO_2	8.85	2288673
Ni-MOF-74-SO_2 variable P 1.2 bar	300	18(5) % Metal bound SO_2, 27(6) % physisorbed SO_2	9.09	2288674
Ni-MOF-74-SO_2 variable P 1.6 bar	300	28(6) % Metal bound SO_2, 32.9(7) % physisorbed SO_2	9.78	2288675
Ni-MOF-74-SO_2 variable P 2.0 bar	300	29(6) % Metal bound SO_2, 32.8(7) % physisorbed SO_2	9.65	2288676

Supplementary Analysis

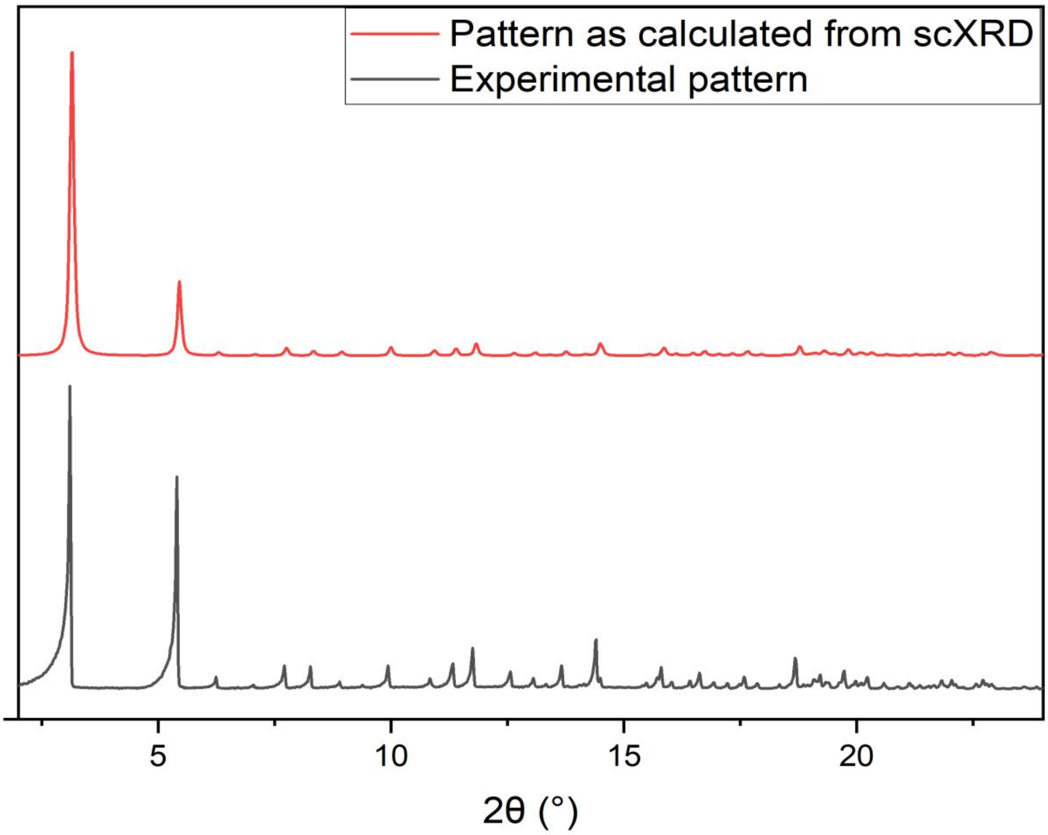

Figure S1: PXRD patterns of Ni-MOF-74 of the experimentally derived structure (black) and calculated from the scXRD modelled structure (red).

Supplementary Table 2: Comparing computed SO_2 binding energies from literature sources.

MOF	-SO_2 binding energy kJ/mol Metal bound	-SO_2 binding energy kJ/mol Non-metal bound	Ref
Mg-MOF-74	89.5	42.6	This work
Mg-MOF-74	91 - 87	N/A	[8]
MIL-160	N/A	60 - 39	[9]
Mg-MOF-74	62	N/A	[10]
Mg-MOF-74	73 - 90	N/A	[11]
Mn-MOF-74	137	N/A	[12]
Mg-MOF-74	78.9	36.6	[13]
Mg-MOF-74	218.8	N/A	[14]

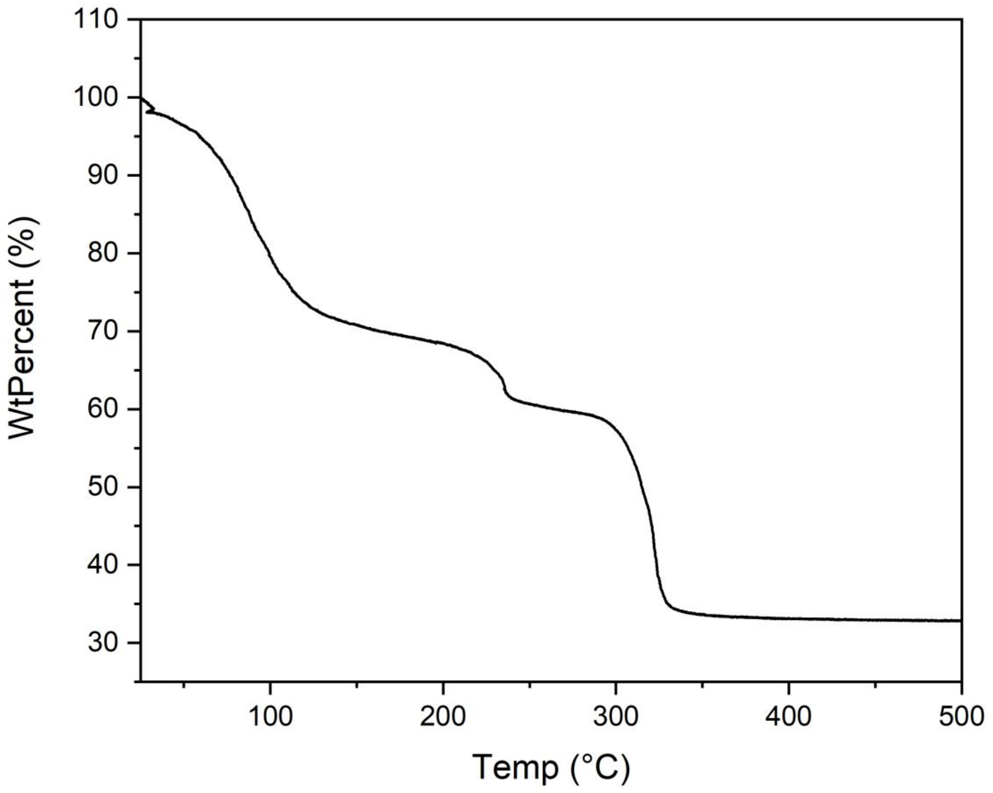

Figure S2: Plot showing thermal gravimetric analysis for Ni-MOF-74 measured in air flow.

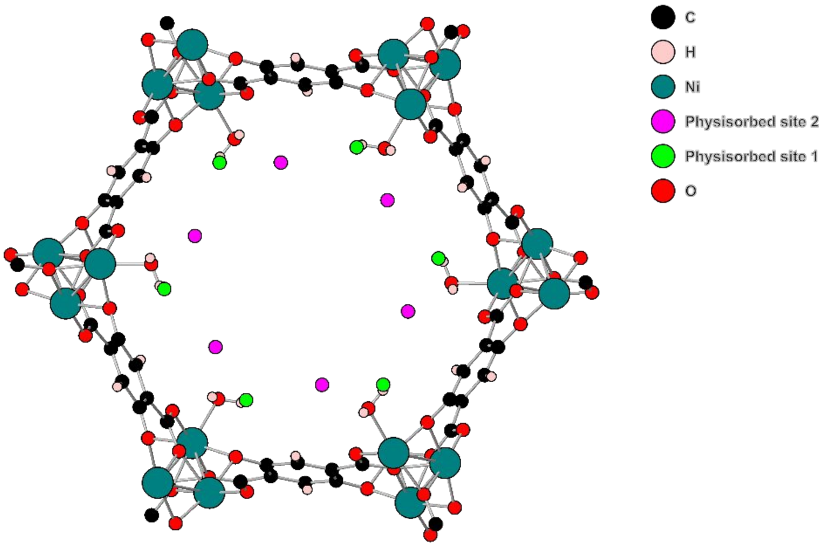

Figure S3: View (ball and sticks) down the crystallographic c-axis of the model used for SO_2 loaded Ni-CPO-27 at 300 K and ≤0.4 bar SO_2.

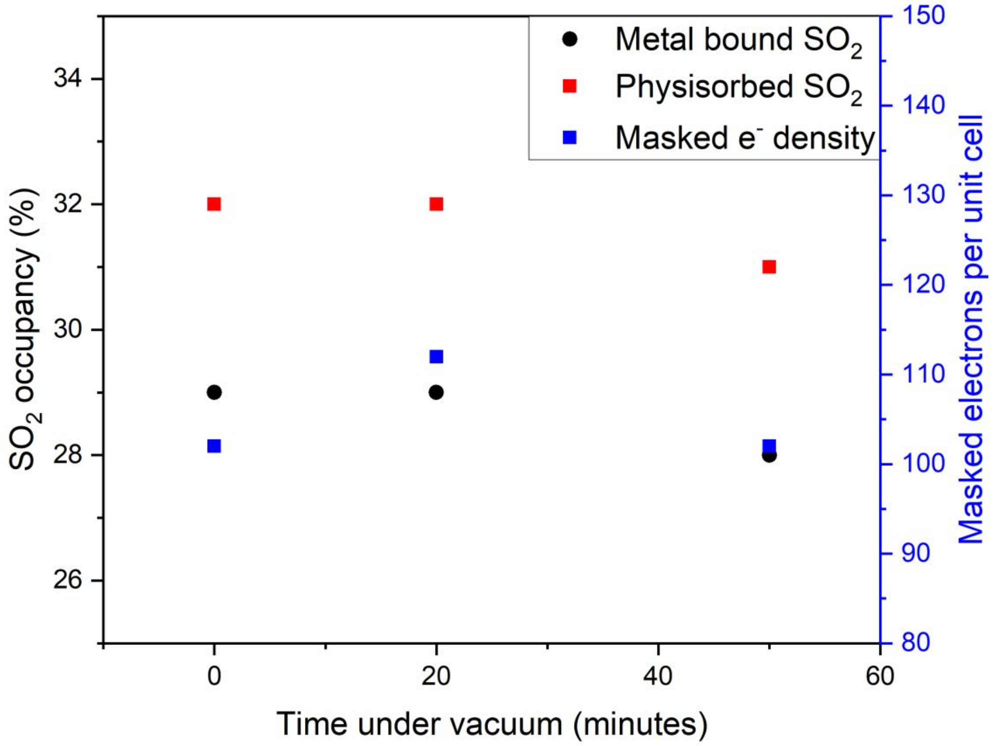

Figure S4: Plot showing how SO_2 loading occupancies of Ni-MOF-74 are affected by application of dynamic vacuum at 300K.

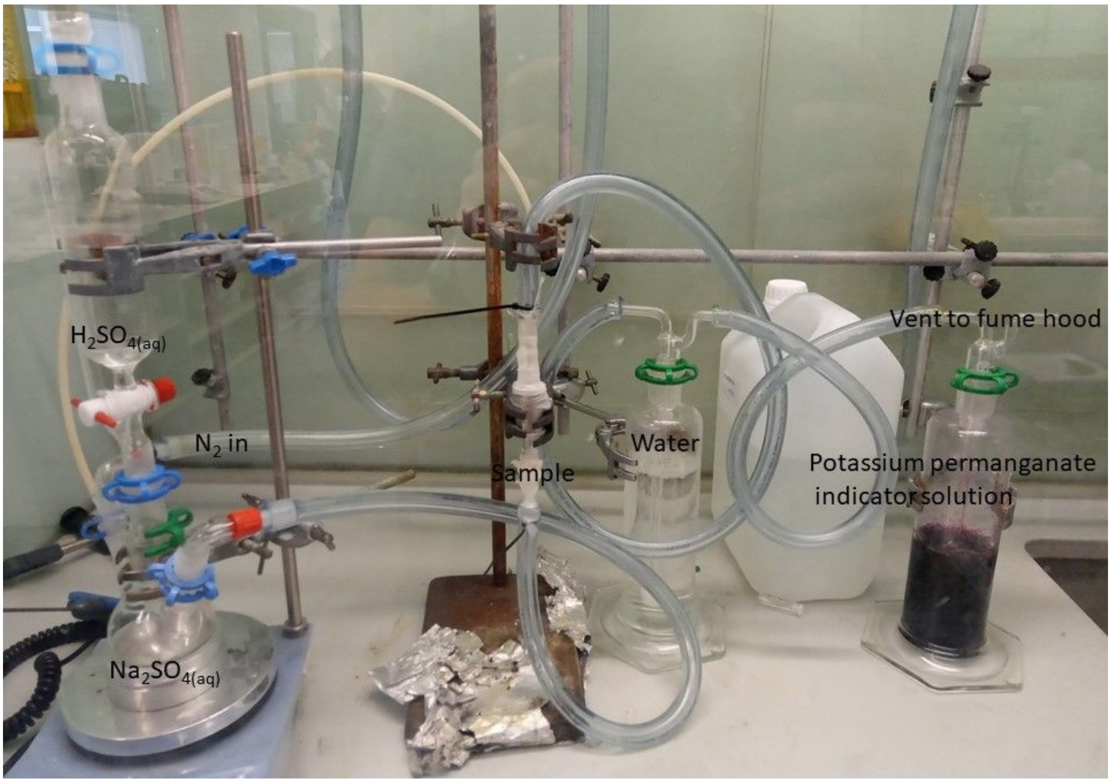

Figure S5: The experimental setup used to load MOFs with SO_2.

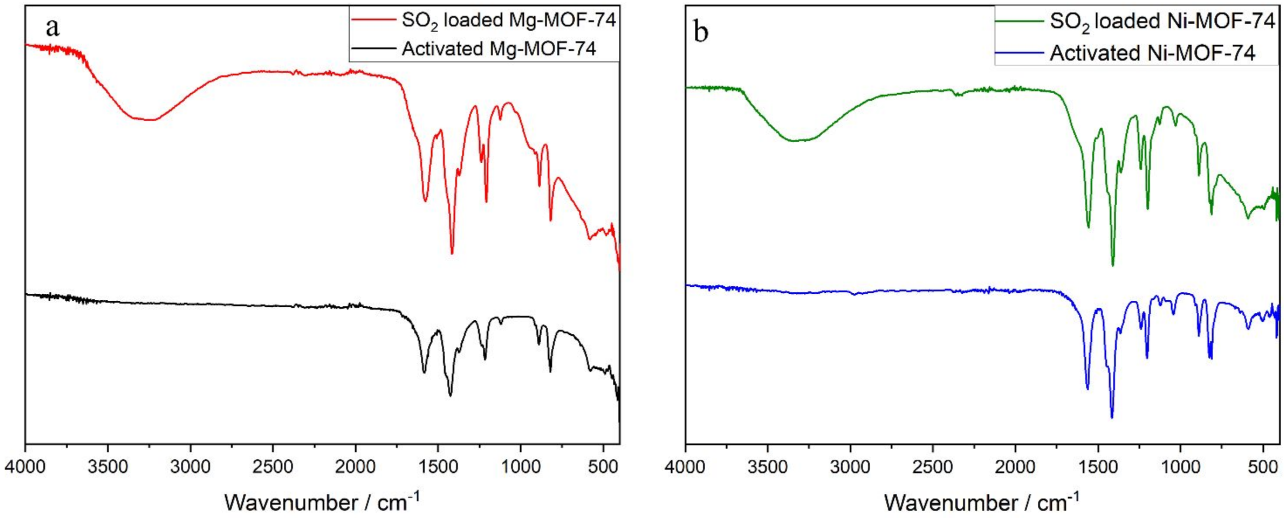

Figure S6: IR spectra before and after SO_2 loading of a) Mg-MOF-74 and b) Ni-MOF-74.

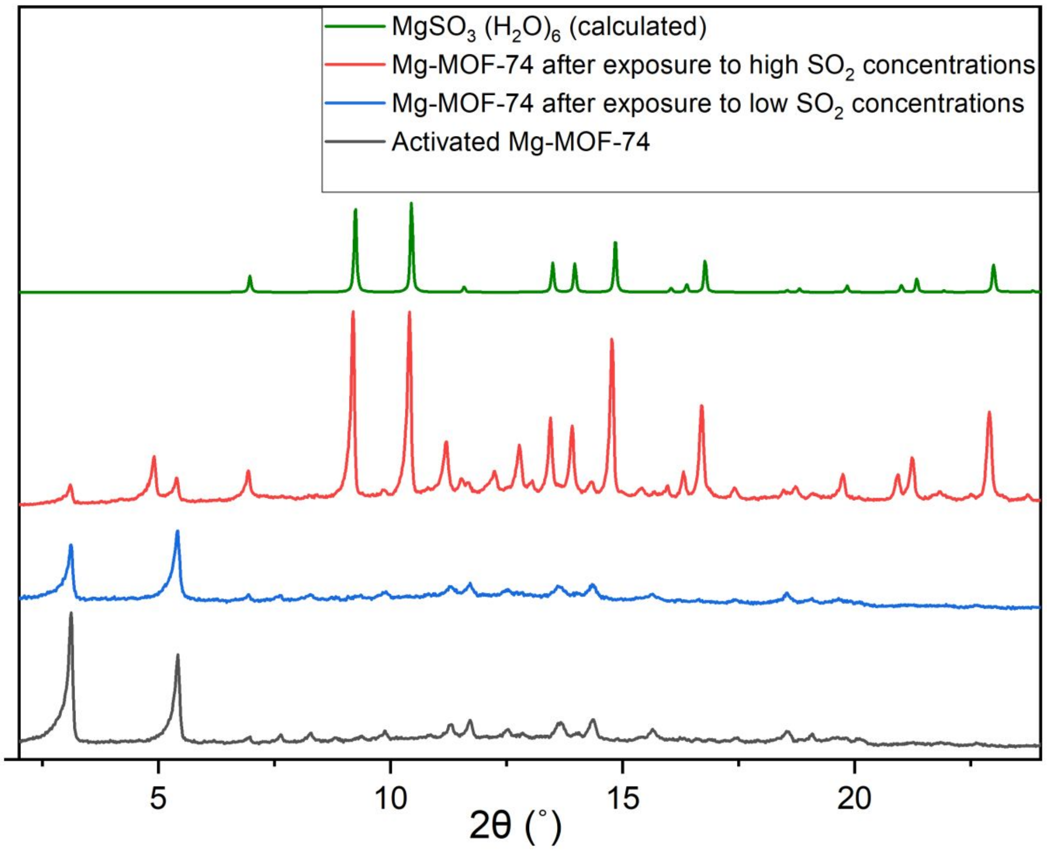

Figure S7: PXRD patterns showing how Mg-MOF-74 behaves under differently concentrated SO_2 gas streams.

Supplementary References

(1) CrysAlisPro, 1.171.41.93a. Rigaku Oxford Diffraction. Rigaku Corporation: Tokyo, Japan 2020.

(2) Winter, G.; Waterman, D. G.; Parkhurst, J. M.; Brewster, A. S.; Gildea, R. J.; Gerstel, M.; Fuentes-Montero, L.; Vollmar, M.; Michels-Clark, T.; Young, I. D.; Sauter, N. K.; Evans, G. DIALS: Implementation and Evaluation of a New Integration Package. *Acta Crystallogr. Sect. D Struct. Biol.* **2018**, *74* (2), 85–97. https://doi.org/10.1107/S2059798317017235.

(3) Dolomanov, O. V.; Bourhis, L. J.; Gildea, R. J.; Howard, J. A. K.; Puschmann, H. OLEX2: A Complete Structure Solution, Refinement and Analysis Program. *J. Appl. Crystallogr.* **2009**, *42* (2), 339–341. https://doi.org/10.1107/S0021889808042726.

(4) Sheldrick, G. M. A Short History of SHELX. *Acta Crystallogr. Sect. A Found. Crystallogr.* **2008**, *64* (1), 112–122. https://doi.org/10.1107/S0108767307043930.

(5) Sheldrick, G. M. Crystal Structure Refinement with SHELXL. *Acta Crystallogr. Sect. C Struct. Chem.* **2015**, *71* (1), 3–8. https://doi.org/10.1107/S2053229614024218.

(6) Gillespie, R. J.; Robinson, E. A. THE SULPHUR–OXYGEN BOND IN SULPHURYL AND THIONYL COMPOUNDS: CORRELATION OF STRETCHING FREQUENCIES AND FORCE CONSTANTS WITH BOND LENGTHS, BOND ANGLES, AND BOND ORDERS. *Can. J. Chem.* **1963**, *41* (8), 2074–2085. https://doi.org/10.1139/v63-299.

(7) Rees, B.; Jenner, L.; Yusupov, M. Bulk-Solvent Correction in Large Macromolecular Structures. *Acta Crystallogr. Sect. D Biol. Crystallogr.* **2005**, *61* (9), 1299–1301. https://doi.org/10.1107/S0907444905019591.

(8) Tan, K.; Zuluaga, S.; Wang, H.; Canepa, P.; Soliman, K.; Cure, J.; Li, J.; Thonhauser, T.; Chabal, Y. J. Interaction of Acid Gases SO2 and NO2 with Coordinatively Unsaturated Metal Organic Frameworks: M-MOF-74 (M = Zn, Mg, Ni, Co). *Chem. Mater.* **2017**, *29* (10), 4227–4235. https://doi.org/10.1021/acs.chemmater.7b00005.

(9) Brandt, P.; Nuhnen, A.; Lange, M.; Möllmer, J.; Weingart, O.; Janiak, C. Metal-Organic Frameworks with Potential Application for SO2 Separation and Flue Gas Desulfurization. *ACS Appl. Mater. Interfaces* **2019**, *11* (19), 17350–17358. https://doi.org/10.1021/acsami.9b00029.

(10) Lee, K.; Howe, J. D.; Lin, L. C.; Smit, B.; Neaton, J. B. Small-Molecule Adsorption in Open-Site Metal-Organic Frameworks: A Systematic Density Functional Theory Study for Rational Design. *Chem. Mater.* **2015**, *27* (3), 668–678. https://doi.org/10.1021/cm502760q.

(11) Yu, K.; Kiesling, K.; Schmidt, J. R. Trace Flue Gas Contaminants Poison Coordinatively Unsaturated Metal-Organic Frameworks: Implications for CO2 Adsorption and Separation. *J. Phys. Chem. C* **2012**, *116* (38), 20480–20488. https://doi.org/10.1021/JP307894E.

(12) Zhang, M.; Huang, X.; Chen, Y. DFT Insights into the Adsorption of NH3-SCR Related Small Gases in Mn-MOF-74. *Phys. Chem. Chem. Phys.* **2016**, *18* (41), 28854–28863. https://doi.org/10.1039/C6CP05557E.

(13) Alonso, G.; Bahamon, D.; Keshavarz, F.; Giménez, X.; Gamallo, P.; Sayós, R. Density Functional Theory-Based Adsorption Isotherms for Pure and Flue Gas Mixtures on Mg-MOF-74. Application in CO2 Capture Swing Adsorption Processes. *J. Phys. Chem. C* **2018**, *122* (7), 3945–3957. https://doi.org/10.1021/ACS.JPCC.8B00938.

(14) Ding, L.; Yazaydin, A. Ö. How Well Do Metal-Organic Frameworks Tolerate Flue Gas Impurities? *J. Phys. Chem. C* **2012**, *116* (43), 22987–22991. https://doi.org/10.1021/JP308717Y.
